# Many Kansas Worksites Offer Few Interventions to Reduce Occupational Sedentary Behavior

**DOI:** 10.3390/ijerph15081745

**Published:** 2018-08-14

**Authors:** Elizabeth Ablah, Elizabeth Grilliot, Hayrettin Okut, Emily L. Mailey, Sara K. Rosenkranz, Richard R. Rosenkranz

**Affiliations:** 1 Department of Preventive Medicine and Public Health, University of Kansas School of Medicine-Wichita; 1010 North Kansas, Wichita, KS 67214, USA; egrilliot@kumc.edu; 2 Office of Research, University of Kansas School of Medicine-Wichita; 1010 North Kansas, Wichita, KS 67214, USA; hokut@kumc.edu; 3 Department of Kinesiology, Kansas State University; Natatorium 8920 Denison Avenue, Manhattan, KS 66506, USA; emailey@k-state.edu; 4 Department of Food, Nutrition, Dietetics and Health, Kansas State University; 212 Justin Hall, 1324 Lovers Lane, Manhattan, KS 66506, USA; sararose@k-state.edu (S.K.R.); ricardo@k-state.edu (R.R.R.)

**Keywords:** sedentary behavior, worksite, workplace, physical activity, occupational health, sedentary lifestyle, exercise, Kansas

## Abstract

The purpose of this study was to identify the extent to which sedentary behavior interventions are being implemented in Kansas worksites. The WorkWell KS Physical Activity Assessment was administered online to 111 worksites across Kansas from October 2016 through April 2018. Each worksite identified a point of contact to complete the worksite-level assessment. Four of the WorkWell KS Physical Activity Assessment’s items assessed interventions that may reduce employees’ sedentary behavior: offering point-of-decision prompts to reduce employees’ sedentary behavior, offering a program for employees to reduce their sedentary time at work, having an organizational norm that allows employees to stand, stretch, and/or move during meetings at least every 30 minutes, and offering standing desks. All 111 worksites that participated in the WorkWell KS Physical Activity Workshop completed the WorkWell KS Physical Activity Assessment, resulting in a 100% response rate. Most worksites (59%, *n* = 65) reported offering no information, program, policy, or environmental change interventions aimed to reduce sedentary behavior. The most commonly reported intervention offered by worksites to reduce employees’ sedentary behavior was standing desks (32%, *n* = 35). Overall, participating worksites reported implementing a few interventions that are designed to reduce sedentary behavior.

## 1. Introduction

Sedentary behavior has been identified as a risk factor, independent of physical inactivity, for several cancers including colorectal, endometrial, prostate, and lung cancer [1,2,3], as well as being a host of other chronic diseases [4]. Sedentary behavior is highly prevalent in office-based workplaces, where many adults spend up to 70 to 80% of their workday being sedentary [5].

Researchers are beginning to explore potentially effective sedentary behavior interventions at a time when multiple-level, multi-strategy interventions (information + environment) are of increasing relevance, particularly in the workplace [6,7]. These comprehensive workplace wellness interventions, adopting best practices at multiple levels of the social ecological model, are capable of creating a culture shift necessary to make an impact [8,9]. Such multiple-level, multi-strategy interventions have demonstrated positive impact associated with implementation and increased employee physical activity [10,11]. Despite the emergence of such interventions, little is known about the extent to which worksites are currently providing interventions to address sedentary behavior among their employees. This paper sought to identify which intervention strategies (information, programs, policy, environmental) are most frequently employed to address sedentary behavior among Kansas worksites.

## 2. Materials and Methods

This study was a cross-sectional survey of WorkWell KS Physical Activity workshop participants. The sample consisted of participants from six workshops (with multiple worksites per workshop), which were conducted in communities across Kansas from October 2016 through May 2017. Kansas worksites’ potential participants were invited by local “champions.” A point of contact, someone knowledgeable of the worksite’s health promotion offerings, was identified for each worksite to complete the physical activity assessment. No incentives were provided, and there was no cost to worksites for participating in the workshop. The University of Kansas School of Medicine-Wichita’s Human Subjects Committee deemed this “not human subjects” research as the data collected were about the worksite.

Prior to participating in a physical activity workshop, teams of worksite representatives participated in a prerequisite workshop, Building the Foundation. Workshops were conducted in the worksite’s community or region. During each workshop, each worksite received a tailored report that outlined which physical activity intervention the worksite was already implementing based on the worksite-level physical activity assessment completed prior to attending the workshop.

### Measures and Analysis

The WorkWell KS Physical Activity Assessment included 34 items, 28 of which assessed physical activity interventions, four items assessed interventions that reduce employees’ sedentary behavior, and two items solicited contact information. The assessment was developed based on best practices to prompt physical activity and promising practices to break up sedentary behavior by standing or moving. The following sedentary behavior interventions were assessed at the worksite level: offering point-of-decision prompts (information) to reduce employees’ sedentary behavior [12], offering a program for employees to reduce their sedentary time at work (e.g., planning details of when, where, how to sit less at work) [13], having an organizational norm that allows employees to stand, stretch, and/or move during meetings, at least every 30 minutes (policy) [14], and offering standing desks (environmental) [15]. Frequencies and percentages were calculated.

Worksites were then categorized into industry classifications based on the North American Industry Classification System (NAICS) and then further stratified into either a sedentary industry group (information; finance and insurance; professional, scientific and technical service; educational service; arts, entertainment and recreation; public administration; and other grant making and giving services) or an active industry group (manufacturing; wholesale trade; and healthcare and social assistance) [16]. Worksites were placed into industry groups based on researcher assumptions, informed by past experience with WorkWell KS worksites. A Chi-Squared with Fisher’s exact tests option was conducted to examine the relationship between industry groups and the presence of interventions to reduce sedentary behavior.

To examine a potential relationship between the number of physical activity interventions implemented and the number of sedentary behavior interventions implemented at each worksite, a Spearman’s Correlation test was conducted.

## 3. Results

Of the 111 worksite teams that participated in a WorkWell KS Physical Activity workshop, 103 had participated in a WorkWell KS Building the Foundation workshop. All 111 worksites completed the WorkWell KS Physical Activity Assessment, demonstrating a 100% response rate. Many worksites (44%, *n* = 49) were small, with 49 or fewer employees; 31% (*n* = 34) had 50 to 249 employees, and 24% (*n* = 27) had 250 or more employees; one worksite did not report the total number of employees. Worksites reported a wide range in the number of employees—from 1 to 3200 (average of 205, and a median of 75) and dependents—0 to 7200 (average of 294, and a median of 84).

Twenty-three percent of worksites (*n* = 26) were located in southeast Kansas, and 21% (*n* = 23) were located in northeast Kansas. Industry type also varied, with most participating worksites representing “educational service” (36%, *n* = 40), 16% (*n* = 18) were from health care and social assistance, 12% (*n* = 13) were from public administration, 7% (*n* = 8) were in grantmaking and giving services, 6% (*n* = 7) were in wholesale trade, 5% (*n* = 6) were in manufacturing, 5% (*n* = 5) were in finance and insurance, 5% (*n* = 5) were in arts, entertainment, and recreation, and the remainder were in information or professional, scientific, and technical services.

Ten percent (*n* = 11) of worksites reported offering a point-of-decision prompt (e.g., e-mail, text, music) prompting employees to move, and 15% (*n* = 17) reported offering a program for employees to reduce the amount of time they sit or are sedentary at work. Eleven percent (*n* = 12) of worksites reported having a policy that allows employees to stand, stretch, and/or move during meetings, at least every 30 minutes; six worksites reported this question as “not applicable, all employees perform manual labor.” Thirty-two percent (*n* = 35) reported offering standing desks.

**Figure 1:**
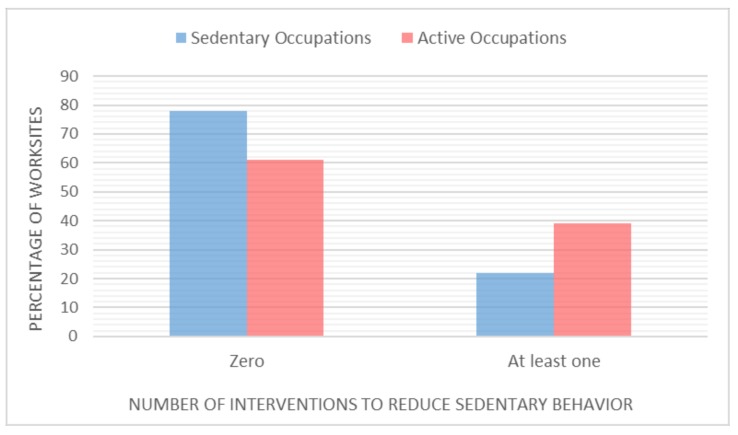
Percentage of worksites offering at least one intervention to reduce sedentary behavior, by industry group.

Most worksites (59%, *n* = 65) reported offering no information, program, policy, or environmental change interventions to reduce sedentary behavior. Over one fifth (23%, *n* = 25) of worksites reported implementing one intervention, 13% (*n* = 14) reported implementing two interventions, 5% (*n* = 6) reported three interventions, and 1% (*n* = 1) reported all four interventions. Of the worksites offering interventions, sedentary occupations (61%) were more likely to offer at least one intervention than active worksites (39%), *p* = 0.036 (Figure 1). There was no difference in the number of interventions implemented by the size of a worksite.

Worksites with interventions to reduce sedentary behavior were correlated with the worksites with interventions intended to increase physical activity, *p* = 0.001.

## 4. Discussion

This study offers an important perspective within sedentary behavior research—there is currently minimal implementation of promising practices to reduce sedentary behavior among a sample of Kansas worksites. As the emerging problem of sedentary behavior has only been recently identified (relative to physical inactivity), interventions to address sedentary behavior are being developed during a period where more researchers are attending to the multiple levels of the socio-ecologic model and identifying the value of policy, systems, and environmental changes (in contrast to individual-level-only interventions). The adoption of upstream interventions (policy and environment) rather than individual-level interventions (information and programs) among participating worksites may be attributable to this shift in emphasis. Although a few worksites have reported implementing interventions to reduce sedentary behavior, the most common intervention was an environmental change- using standing desks. As this study is the first to assess worksite-level sedentary interventions, there are no existing data to compare the current findings to other worksites in Kansas, in other states, or at a national level. Existing worksite-level data consists of singular interventions, implemented at a single time point, such as the four sedentary behavior interventions we measured collectively, point-of-decision prompts (information intervention) [11], offering a program for employees to reduce their sedentary time at work [12], having an organizational norm that allows employees to stand, stretch, and/or move during meetings (policy intervention) [13], and offering standing desks (environmental intervention) [14]. While each has shown promise to reduce sedentary behavior in worksites, a combination of all the interventions, a multi-component, comprehensive intervention, has not been assessed. Future studies’ use of worksite-level assessments, even when used in conjunction with individual-level assessments of sedentary behavior, could advance the implementation and evaluation of worksite wellness efforts.

The current study’s strength is its approach-surveying employers about the interventions they offer to their employees. Measures of individual employees’ sedentary behavior can be timely and resource-intensive. A simple assessment that inventories employer-provided interventions that reduce employees’ sedentary behavior provides a clear insight into the causes of and solutions to sedentary behavior at the worksite. We are not aware of other existing worksite-level assessments that solely address sedentary and/or physical activity levels at the worksite. More research is needed to explore the most cost- and time-effective interventions that worksites can implement to reduce sedentary behavior. Despite finding sedentary occupations were more likely to offer at least one sedentary behavior intervention than active occupations, more research is warranted to identify specific strategies for sedentary worksites to launch additional interventions, as their employees are more likely to experience increased health risks associated with sedentary behavior. Additionally, future research is warranted to explore the effectiveness of various interventions and their associated levels of work performance, productivity, and health outcomes.

### Limitations

The current study’s weaknesses include the small sample size (limiting external validity), use of self-report measures (limiting internal validity), stratification of worksites into industry groups by researchers (based on assumption), and potential selection bias (participating worksites might have been more receptive to an invitation to participate). Despite the fact that this was a convenience sample, worksites reported implementing a few interventions to reduce sedentary behavior. Although the assessment was to be completed by employees who were most aware of the worksites’ health promotion offerings, it was completed by one person (or a group of employees) at each worksite. These limitations are likely to reduce the generalizability of current findings.

## 5. Conclusions

This study suggests that promising practices for sedentary behavior interventions are not largely being implemented at the participating worksites, and potentially across Kansas. One of the strongest interventions known to date, the use of standing desks, was the most prevalent intervention reported by participating worksites. Nonetheless, most worksites reported offering no sedentary behavior interventions to employees. Such organizational inaction may inadvertently contribute to promoting sedentary behavior and chronic diseases.
